# Study protocol for “Psilocybin in patients with fibromyalgia: brain biomarkers of action”

**DOI:** 10.3389/fpsyt.2024.1320780

**Published:** 2024-06-04

**Authors:** Julia Bornemann, James B. Close, Kirran Ahmad, Tommaso Barba, Kate Godfrey, Lauren Macdonald, David Erritzoe, David Nutt, Robin Carhart-Harris

**Affiliations:** ^1^ Centre for Psychedelic Research, Department of Brain Science, Imperial College London, London, United Kingdom; ^2^ Psychedelics Division, Neurology, Psychiatry and Behavioural Sciences Weill Institute for Neurosciences, University of California, San Francisco, San Francisco, CA, United States

**Keywords:** psilocybin, psychedelic therapy, chronic pain, fibromyalgia, EEG

## Abstract

**Background:**

Chronic pain is a leading cause of disability worldwide. Fibromyalgia is a particularly debilitating form of widespread chronic pain. Fibromyalgia remains poorly understood, and treatment options are limited or moderately effective at best. Here, we present a protocol for a mechanistic study investigating the effects of psychedelic-assisted-therapy in a fibromyalgia population. The principal focus of this trial is the central mechanism(s) of psilocybin-therapy i.e., in the brain and on associated mental schemata, primarily captured by electroencephalography (EEG) recordings of the acute psychedelic state, plus pre and post Magnetic Resonance Imaging (MRI).

**Methods:**

Twenty participants with fibromyalgia will complete 8 study visits over 8 weeks. This will include two dosing sessions where participants will receive psilocybin at least once, with doses varying up to 25mg. Our primary outcomes are 1) Lempel-Ziv complexity (LZc) recorded acutely using EEG, and the 2) the (Brief Experiential Avoidance Questionnaire (BEAQ) measured at baseline and primary endpoint. Secondary outcomes will aim to capture broad aspects of the pain experience and related features through neuroimaging, self-report measures, behavioural paradigms, and qualitative interviews. Pain Symptomatology will be measured using the Brief Pain Inventory Interference Subscale (BPI-IS), physical and mental health-related function will be measured using the 36-Item Short Form Health Survey (SF-36). Further neurobiological investigations will include functional MRI (fMRI) and diffusion tensor imaging (changes from baseline to primary endpoint), and acute changes in pre- vs post-acute spontaneous brain activity – plus event-related potential functional plasticity markers, captured via EEG.

**Discussion:**

The results of this study will provide valuable insight into the brain mechanisms involved in the action of psilocybin-therapy for fibromyalgia with potential implications for the therapeutic action of psychedelic-therapy more broadly. It will also deliver essential data to inform the design of a potential subsequent RCT.

## Introduction

Chronic pain is a leading cause of disability worldwide (1, 2). Fibromyalgia (FM) is a particularly debilitating form of chronic generalized pain (3–5) with reported prevalence rates varying from between 0.4 and 8% (6–10) FM is characterised by widespread pain, fatigue, sleep difficulties and cognitive disturbance including memory and ability to concentrate e.g., brain fog (5). Frequently reported concomitant symptoms are irritable bowel syndrome (IBS), headache, and temporomandibular disorder (6, 11). Compared with other types of chronic pain, people with FM exhibit disproportionately high rates of psychological comorbidity (3, 9) with 60-80% also experiencing comorbid depression and/or anxiety (12–14). Additionally, FM populations present markedly high rates of lifetime (15), and particularly childhood trauma (16, 17). Women are significantly more likely to be affected than men (8, 11, 18).

The aetiology and pathology of FM remain poorly understood (19), although current hypotheses centre around combined immunological (20), psychological (21), stress (21) and trauma-related (22, 23) mechanisms. Certain physiological changes are regularly observed in FM populations, including a hyperexcitable central nervous system via high levels of glutamate, as well as additional dysregulated monoamine neurotransmitter expression, particularly of serotonin and dopamine (23). Still, this limited understanding has resulted in relatively few effective treatment options. First line medical intervention aims to address these molecular changes and commonly includes off-label anti-depressants [e.g., Tricyclic Antidepressants (TCAs), Selective Serotonin Reuptake Inhibitors (SSRIs), and Serotonin and Norepinephrine Reuptake Inhibitors (SNRIs)], anticonvulsants (e.g., gabapentin, pregabalin), and opioids (e.g., tramadol) (23, 24), while non-pharmacological options involve physiotherapy, pain-management programmes, and cognitive behavioural therapies (24). While these treatment options have shown results in other specific conditions, their efficacy in FM populations is thought to be moderate at best (25–28). Importantly, even if patients report a decrease in pain, their comorbid mental health symptoms are often left insufficiently addressed (25). Neglecting this essential aspect of the chronic pain experience is especially problematic due to the known bi-directional relationship between pain perception and mental health (2, 29, 30). FM is a highly disabling and growing problem worldwide. The lack of adequate treatment is especially problematic for chronic conditions such as FM where quality of life is severely impacted, thus, novel, and integrative treatment options are urgently needed.

The past decade has witnessed a “renaissance” in clinical psychedelic research. Classic psychedelic drugs include LSD (lysergic acid diethylamide), psilocybin (the active compound in “magic mushrooms”), and DMT (dimethyltryptamine, the active compound in the *ayahuasca*). A growing body of data suggests the safety (31) and efficacy of psychedelics in clinical populations including depression (32–34), addiction (35, 36), obsessive compulsive disorder (37), and end-of-life distress (38–41), as well as in healthy populations (42, 43).

Classic psychedelics act primarily through agonism of excitatory serotonin 2A receptors, though 5-HT 1A, 1C, and 2C receptors agonism is also observed, as well as indirect dopaminergic action (31). Activation of serotonin 2A receptors appears to dysregulate population-level spontaneous neural oscillations (44–46) which may subsequently account for increases in markers of anatomical neuroplasticity (47–51). Increased plasticity via serotonin 2A agonist psychedelics may increase an individual’s sensitivity to extra pharmacological contextual factors known to guide therapeutic outcomes via psychotherapy (52).

In a therapeutic context, as seen in psychedelic-assisted therapy (PAT), it is theorised that psychedelics could open a window of plasticity that might facilitate the reappraisal of deeply entrenched, maladaptive thought patterns towards therapeutically useful outcomes (52–54). Such increases in psychological flexibility make FM is a particularly attractive target for investigation due to its hallmark psychological rigidity (55) and significant cross-over with depression which is potentially positively impacted by PAT (32, 33). Antidepressant action may also be supported by the abovementioned modulation of monoamine neurotransmitters (31).

While psychedelics have not yet been investigated in a FM context, historical studies suggest potential action in chronic pain conditions including cancer pain (56–60) and phantom limb pain (61–63). Interest has recently re-emerged, with modern work suggesting psychedelics’ anti-inflammatory action (63–65) and potential efficacy in treating headache disorders (66–68), phantom limb pain (69), and acute pain (70). Indeed, proposed, and ongoing studies investigating the effects of classic psychedelics in phantom limb pain (71), lower back pain (72), and fibromyalgia (73) are likely to advance our understanding in this area.

## Patient and public involvement

A growing body of ‘grey’ literature of case reports and/or protocols pertaining to psychedelic-use has emerged online on forums such as Reddit, Bluelight, and Erowid. A significant portion of these relates to self-medication for chronic pain (74). Specific, crowd-sourced protocols have emerged as a result; organisations such as Clusterbusters boast over 10,000 members who follow published guidance for psychedelic self-medication for cluster headaches (75). Such anecdotal reports may inspire hypotheses for researchers designing research studies and clinical trials. Indeed, Schindler et al. formally investigated the viability of psychedelics on cluster headaches following the Clusterbusters protocol (68). Accordingly, we sought to learn from the lived experience of people who have self-medicated with classical psychedelics for chronic pain to aid in the design of the present study (see Methods section).

## Overview/aims

This paper presents the protocol for a mechanistic study investigating the effects of PAT in a fibromyalgia population. The principal focus of this trial is the central mechanism(s) of psilocybin i.e., in the brain and on associated mental schemata, primarily captured by electroencephalography (EEG) recordings of the acute psychedelic state. Secondary outcomes will aim to capture broad aspects of the pain experience and related features through Magnetic Resonance Imaging (MRI), self-report measures, behavioural paradigms, and qualitative interviews. This study aims to serve as a preliminary investigation into potential mechanisms of psilocybin in this study population. By publishing our protocol before commencing data collection, we aim to contribute towards a scientific culture of openness and rigor.

## Methods and analysis

Here we describe a single arm, fixed sequence, single-blind, within-subjects study. Our primary outcomes investigate psychological flexibility through potential neurophysiological markers (specifically Lempel-Ziv complexity (LZc) recorded acutely using EEG, and the (Brief Experiential Avoidance Questionnaire (BEAQ) measured at baseline and primary endpoint). Secondary outcomes will provide a context to complement these mechanistic EEG data. Pain symptomatology will be measured using the Brief Pain Inventory Interference Subscale (BPI-IS) and broader aspects of health, including physical and mental health functioning will be measured using the 36-Item Short Form Health Survey (SF-36). We will additionally collect self-reported data on the strength of personally held negative beliefs in line with the relaxed beliefs under psychedelics model (55).

Further neurophysiological investigations will include functional magnetic resonance imaging (fMRI and DTI) (changes from baseline to primary endpoint), and EEG (changes from baseline to in putative resting state and ERP plasticity markers. We believe that a breakthrough on the brain mechanisms involved in therapeutically relevant change process catalysed by psilocybin, will have broad and important scientific and clinical implications.

Patient and public involvement (PPI) was used throughout the process of trial preparation and contributed to protocol development. PPI is an emerging research method across mental health and within psychedelic research (76, 77). The method aims to produce research “with” rather than “for” people with lived experience. Such co-created research fosters empowerment and trust in research produces relevant and transparent outputs (78).

PPI informed our therapeutic protocols, inspired further research questions, and resulted in the development of novel measures investigating the somatic elements of the psychedelic experience [see Bornemann et al. (79)]. Further, our panel of patient contributors has provided input to, and approved all patient facing documents.

### Recruitment

We will recruit up to twenty participants with fibromyalgia as defined by the American Rheumatological Society 2016 diagnostic criteria (80). Study completion is set as completion of the final study visit (primary endpoint). Full entry criteria are outlined in **Table 1**.

**Table 1:** Key In-/Exclusion criteria.

Inclusion criteria
Fibromyalgia Syndrome as diagnosed by an appropriate medical professional using the American College of Rheumatology diagnostic criteria, lasting for more than 3 months.
Over 18 years of age
UK resident registered with a primary care medical practice
Sufficiently competent in English with capacity to provide written informed consent
Agreement for research team to contact primary and/or secondary care team over the course of the study
No psychedelic use in the past 6 months

Exclusion criteria
Current or previously diagnosed psychotic disorder, bipolar disorder, or mania
Immediate family member with a diagnosed psychotic disorder
History of serious suicide attempts
Currently using medication which could interact with psilocybin including anti-psychotics, mood stabilizers & serotonergic antidepressants including SSRIs, SNRIs, and TCAs.*
Actively enrolled on pain management programme over course of study or awaiting further investigations for pain
On waiting list for interventional treatment for pain (e.g. surgery or targeted injections) Medical contraindications e.g., epilepsy, migraine, focal scalp sensitivity
MRI contraindications (e.g. metal implants)
Blood or needle phobia
People who are pregnant, planning to get pregnant, or currently breastfeeding
Unable to engage with physical demands of dosing session (i.e. attend centre and remain in research facility for an extended period of time)
Unable to access virtual meetings/phone for remote follow-ups
Limited life expectancy (<18 months)
Patients consuming more than 35 units of alcohol per week

*Antipsychotic medications and mood stabilisers may attenuate the effects of psilocybin. In this population, people may be using these medications as adjunctive treatments for coexisting depression, anxiety, and also for sleep. Antipsychotic medications are contraindicated and thus those on antipsychotic medication (e.g., olanzapine) will not be eligible to take part in this study. The same is true for mood stabilizing mediations and many antidepressant medications with serotonergic action, including SSRIs, SNRIs, and TCAs. Therefore, we will not be recruiting patients currently prescribed any antidepressant medication, with the exception of Bupropion, which is not serotonergically active.

Recruitment will take place via flyers, word-of-mouth, and from a pool of self-referrals submitted via a secure centralised e-mail address using a standardised referral form. Participant information sheets will be openly accessible on our study website (81). Primary care providers will be contacted to confirm eligibility.

### Study visits

**Figure 1:**
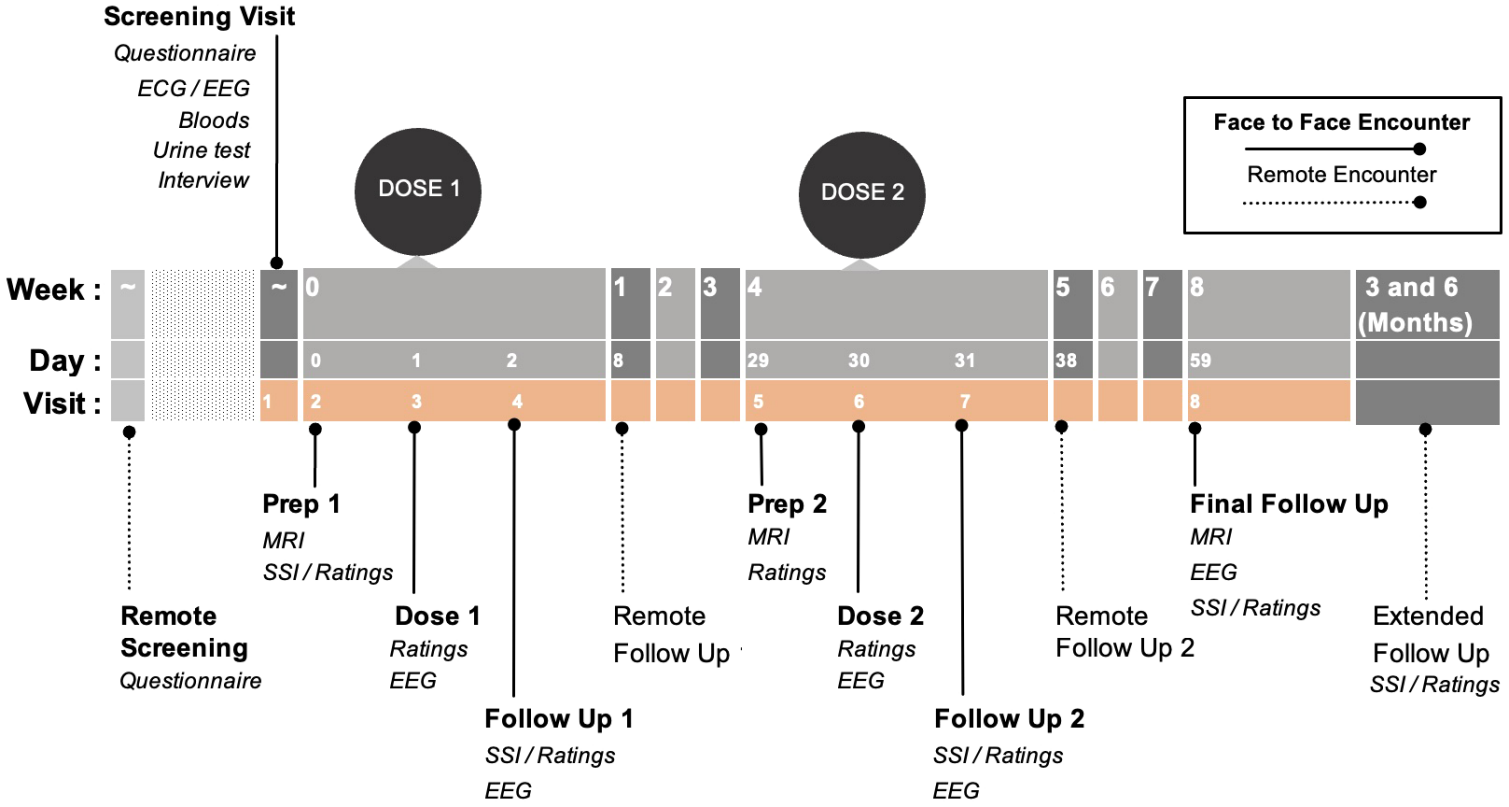
Study timeline: Pink background represents the active study period. Dotted lines represent remote encounters. Please note: this dosing figure is intentionally left blinded. Researchers interested in the specific dosing protocol are welcome to contact the communicating authors for additional information. ECG: Electrocardiogram; EEG: Electroencephalogram; MRI: Magnetic Resonance Imaging; SSI: Semi-structured Interview.

Participants will attend eight study visits (screening, two preparation sessions, two dosing sessions, and three follow-up sessions) over the eight-week study period (see **Figure 1**). Participants will attend two dosing sessions and receive psilocybin at least once (up to 25mg). Dosing sessions are separated by four weeks. Long-term follow-ups will be collected for six-months post study completion, with two remote in-depth check-ins at three and six months. Researchers interested in the specific dosing protocol are welcome to contact the communicating authors for additional information.

#### Screening and consent

Eligible self-referrals will be invited to a remote screening call where informed consent to begin the screening interviews will be collected. Remote screening calls serve to provide information to participation and determine initial eligibility.

If the participant passes initial eligibility at the remote screening, they are invited to a screening visit. Following a thorough explanation of the study and screening process, patients will have the opportunity to ask questions before providing full written informed consent to participate in the study. They will then have a physical exam, psychiatric assessment including MINI, electrocardiogram (ECG), urine samples and blood sample for routine blood testing. Baseline EEG resting state measures will be collected. This will also serve as an EEG tolerability test.

Information regarding the screening/enrolment process including retention and demographics will be published upon study completion.

#### Psychedelic assisted therapy

This study investigates the effects of PAT. While no single unifying PAT protocol exists, common principles are followed across research and will be employed here. Study visits will take place in a comfortable, low-lit environment (82). As comfort allows, patients will be in semi-reclined positions throughout. Music is known to powerfully affect PAT (83); as such, playlists were carefully co-created with music therapists.

As is standard across psychedelic studies, the therapeutic approach is largely self-directed (84) and is informed by humanistic approaches (85). Of particular interest in this context is Acceptance and Commitment Therapy (ACT), a widely used modality in pain-management (28, 86, 87), and an approach increasingly explored for PAT protocols (88–90). We have integrated various aspects of established pain management practices to accommodate the complex needs of our population. Two “guides” will support each participant for the duration of the trial. Guides will prepare the patient for the experience in remote and in-person preparation sessions. Session objectives will be standardised and include areas such as psychoeducation, trust building, and intention setting. The therapeutic manual for this study will be published once data collection is complete. Patients will also have an MRI scan at each preparation visit with visits lasting approximately 4 hours.

Dosing days will occur the day after preparation sessions. Patients will spend ~8 hours at the research facility, with the drug effect lasting for approximately 4-6 hours. Patients will be wearing eye masks and headphones, as comfort allows. EEGs will be recorded before, and during the acute drug state. An on-site medic will ensure participant safety throughout the day and will approve patients before their discharge at the end of the day. Local participants have the option of spending the night at home if accompanied by a trusted person; all participants may stay at provided on-site accommodation.

Integration days will occur the morning after the dosing day. Participants will meet with their guides to discuss their experience and complete EEG recordings. The visit lasts ~4 hours. Guides will check in with participants at remote follow-ups 1 week later as well. The final follow-up will run as the other integration sessions but will also include one final MRI. Patient-reported outcome measures will be collected remotely after each study visit, as well as remotely after primary study endpoint.

### Safety, monitoring, and reporting procedures

Participant safety was, and will continue to be, the guiding principle for all study related decisions. Participants will always be chaperoned when under the care of the study team, including medical supervision on dosing days where discharge may only occur once the doctor on site has deemed it safe for the participant to leave. Patient wellbeing will additionally be assessed the next morning, and the following week.

Participant mood and pain scores will be monitored continually from enrolment to the end of the 6-month follow up period. Automatic alerts will immediately alert the study team of any reports of thoughts of self-harm or suicide. These will be escalated to therapy and medical teams and followed up with as appropriate. All adverse events will be reported.

### Outcome measures

We will collect neurophysiological, self-reported, qualitative, and behavioural data. Please see **Tables 2**–**4** for a summary of all outcome measures. Please refer to **Table 5** for a breakdown of primary and secondary outcomes.

**Table 2:** Summary of patient-reported predictor, patient impression, and good clinical practice measures.

Measure	Screen	Remote Baseline	Prep 1	Dosing Day 1		Integration 1	Prep 2	Dosing Day 2		Integration 2	Final Follow Up	Extended Remote Follow Ups		
				Pre-Dose	Post-Dose			Pre-Dose	Post-Dose			Monthly	3 Month	6 Month
***Week***	~	-1	1	1		1	4	4		4	8	~		
***Day***	~	-7	0	1		2	29	30		31	59	~		
***Visit***	1	~	2	3		4	5	6		7	8	~		
Baseline Static														
*Demographics*	x													
*PainDETECT Phenotyping*	x													
Predictors														
*The Modified Tellegen Absorption Scale (MODTAS)*		x												
*The Short Suggestibility Scale (SSS)*		x												
*Stanford Expectations of Treatment Scale (SETS)*		x												
*Psychedelic Predictor Scale*			x					x						
Patient Impression Measures														
*Patient Global Impression Of Change Score (PGIC)*										x	x			x
*Perceived Treatment Questionnaire*						x				x	x			
*Scale to Assess Therapeutic Relationship (STAR) (Patient Version)*			x				x							
Good Clinical Practice Measures (Guides only)														
*Scale to Assess Therapeutic Relationship (STAR)(Clinician Version)*			x		x	x	x		x	x	x			
*Adverse Events & Serious Adverse Events Record*			x		x	x	x		x	x	x	x	x	x

**Table 3:** Summary of patient-reported sub-acute and acute change measures.

*Measure*	*Screen*	*Remote Baseline*	*Prep 1*	*Dosing Day 1*		*Integration 1*	*Prep 2*	*Dosing Day 2*		*Integration 2*	*Final Follow Up*	*Extended Remote* *Follow Ups*		
				**Pre-Dose**	**Post-Dose**			**Pre-Dose**	**Post-Dose**			**Monthly**	**3 Month**	**6 Month**
***Week***	~	-1	1	1		1	4	4		4	8	~		
***Day***	~	-7	0	1		2	29	30		31	59	~		
***Visit***	1	~	2	3		4	5	6		7	8	~		
***Sub-Acute Change Measures***														
*Brief Pain Inventory Short Form*	x	x	x	x		x	x			x	x	x	x	x
*Single Item Sleep Score*			x	x		x	x	x		x	x			
*9-Item Patient Health Questionnaire (PHQ-9)*		x									x	x	x	x
*Warwick-Edinburgh Mental Wellbeing Scale (WEMWBS)*		x									x	x	x	x
*7-Item Generalised Anxiety Disorder Questionnaire (GAD-7)*		x									x	x	x	x
*Brief Experiential Avoidance Questionnaire (BEAQ) *		x									x	x	x	x
*Snaith-Hamilton Pleasure Scale (SHAPS)*		x				x				x	x			
*Chronic Pain Self-Efficacy Scale (CPSS)*		x									x			x
*Watts Connectedness Scale (WCS) *		x									x			
*Function of Self-Criticizing/Attacking Scale (FSCS)*		x									x			
*The Self Experiences Questionnaire (SEQ)*		x									x			
*8-item Committed Action Questionnaire (CAQ-8)*		x									x			x
*8-item Chronic Pain Acceptance Questionnaire (CPAQ-8)*		x									x			x
*Fibromyalgia Impact Questionnaire (FIQ)*		x									x			x
*Multidimensional Assessment of Interoceptive Awareness (MAIA)*		x									x			x
*Pain Catastrophizing Scale (PCS) *		x				x				x	x			x
*Experiences in Close Relationships Questionnaire (ECR-M16)*		x									x			
*Psychological Inflexibility in Pain Scale (PIPS)*		x									x			x
*Cognitive Fusion Questionnaire (CFQ)*		x									x			x
*Metaphysical Beliefs Scale (Self-Constructed)*		x									x			x
*Somatic Processing Questionnaire (Self-Constructed)*						x				x				
*The Centrality of Events Scale (Short Version)*						x				x	x			x
*Multifaceted Psychological Integration Assessment (M-PIA)*		** **				x				x	x	x		x
***Dosing Day Change Measures***														
*The Emotional Breakthrough Inventory (EBI) *					x				x					
*Setting Questionnaire (SQ, self-constructed)*					x				x					
*11 Dimension Altered States of Consciousness Scale (11D ASC) *					x				x					
*The Challenging Experience Questionnaire (CEQ)*					x				x					
*The Imperial Overview Item*					x				x					
*Geneva emotional music scales (GEMS)*					x				x					
*Awe Experiences Scale*					x				x					
*Psychological Insight Questionnaire*					x				x					
*States of Mindfulness Scale (SMS) Body Sub-Scale *					x				x					
*Relaxed and Embodied Beliefs Questionnaire (Self Constructed)*			x		x				x		x			
*Embodiment Items (Self-Constructed)*		** **			x				x		x			x
*Expectation item (self-constructed)*		x		x				x						

**Table 4:** Summary of all other data types.

*Measure*	*Screen*	*Prep 1*	*Dosing Day 1*			*Integration 1*	*Prep 2*	*Dosing Day 2*			*Integration 2*	*Final Follow Up*	*Remote Extended Follow Up*
			**Pre-Dose**	**+90 mins**	**+150 mins**			**Pre-Dose**	**+90 mins**	**+150 mins**			**6 Months**
***Week***	~	1	1			1	4	4			4	8	~
***Day***	~	0	1			2	29	30			31	59	~
***Visit***	1	2	3			4	5	6			7	8	~
													
***EEG***	** **	** **	** **	** **	** **	** **	** **	** **	** **	** **	** **	** **	
*Tolerability Test*	x												
*Resting State Eyes Closed*	x		x	x	x	x		x	x	x	x	x	
*Resting State Eyes Open*	x		x	x	x	x		x	x	x	x	x	
*rMMN*						x					x		
*vLTP*						x					x		
*Heart Rate Discrimination Task*						x					x		
													
***MRI***													
*Structural*		x					x					x	
*Functional*		x					x					x	
													
***Behavioural***													
*Oura Ring (Ongoing)*	x	x	x	x	x	x	x	x	x	x	x	x	
*Heart Rate Discrimination Task*						x					x		
													
***Qualitative***													
*Unstructured Interview*	x												
*Semi-structured interview*		x				x					x	x	x

**Table 5:** Summary of timepoints for analysis.

PsiloPain Outcome Measures	
*Measure*	Timepoints for Analysis
Primary Outcome Measures	
Neurophysiology	
*Lempel-Ziv complexity (LZc) (EEG)*	DD1 vs DD2
*Modularity (MRI)*	Prep 1 vs Prep 2 vs FFU
Secondary Outcomes	
Neurophysiology	
*Plasticity via vLTP paradigm (EEG)*	FU1 vs FU2
*Predictive Processing via rMMN (EEG)*	FU1 vs FU2
*Alpha power (EEG)*	DD1 vs DD2
*DTI (MRI)*	Prep 1 vs Prep 2 vs FFU
Physiology	
*Heart rate Variability (Oura)*	4 weeks Post-Dose 1 vs 4 weeks Post-Dose 2
*Sleep Quality (Oura)*	4 weeks Post-Dose 1 vs 4 weeks Post-Dose 2
*Interoception (Heartrate Discrimination Task)*	FU1 vs FU2
Primary Patient Reported Outcome Measures	
*Brief Experiential Avoidance Questionnaire (BEAQ)*	Baseline to primary endpoint
Secondary Patient Reported Outcome Measures	
*Core Pain Outcomes*	FU1 vs FU2
*Acute Mediators*	DD1 vs DD2
*Wellbeing/Mental Health Outcomes*	FU1 vs FU2
*Qualitative*	
*Interviews*	Prep 1 vs FFU

#### Mechanistic neurophysiology measures

The present study is conceived as an early-phase mechanistic study, intended to investigate the central action of psychedelics in a population of people living with fibromyalgia. EEG will be recorded at 6 visits: screening, Dose 1, Integration 1, Dose 2, Integration 2, and Final Follow Up (see **Table 3**).

The primary outcome measure is Lempel-Ziv Complexity (LZc) of the resting state EEG signal during dosing days as detailed above. LZc is a compressibility algorithm used to measure signal diversity and has been previously investigated in psychedelic contexts (91–93). Here we hypothesise an increase in LZc under psilocybin.

In addition to resting state, participants will complete the visual Long-Term Potentiation (vLTP) (94) and roving Mismatch Negativity (rMMN) (95, 96) tasks at integrations to investigate the post-acute neuroplasticity and predictive coding. We also hypothesise reduced alpha power under psilocybin.

We will complement these measures with structural and functional MRI data. We will collect MRI data at 3 timepoints: Prep 1, Prep 2, and Final Follow Up (see **Table 3**). We will investigate changes in resting-state activity and connectivity, as well as structural changes including diffusion imaging. We hypothesise decreases in brain network modularity after PAT and decreases in diffusivity in prefrontal to subcortical region white-matter tracts, indexed by the diffusion imaging.

#### Patient reported outcome measures

While the primary interest of the study is in the neural mechanisms involved in psychedelic-mediated action in a fibromyalgia population, we will also collect efficacy measures at every timepoint of the study. Patient-reported outcome measures (PROMs) will be collected in-person and remotely (via video calls and the online survey platform “Alchemer”). All baseline measurements will be taken at Prep 1, except for Symptom Severity Score and the Widespread Pain Index which will be collected at the Screening Visit. Baseline EEG and MRI will be collected at screening and Prep 1 visits, respectively. All primary endpoint measurements (including EEG and MRI) will be collected at final follow up (8 weeks after prep 1). Please see **Tables 2**, **3** for a full list of PROMs.

PROMs are grouped as “Core Pain Outcomes,” “Wellbeing/Mental Health Outcomes,” or “Acute mediators” (see table). We will also be collecting “Patient Impression Measures” to assess the effects of expectancy and blinding efficacy. We will assess the therapeutic relationship using the STAR outcome measure, filled in by both patients and their guides. We hypothesise improvements in Core Pain Outcomes and Wellbeing/Mental Health Outcomes.

#### Behavioural and physiological

We will collect 2 other types of behavioural data investigating interoception and physiology (see **Table 4**). Changes in interoceptive accuracy will be measured using the Heartrate Discrimination Task (HRD) (97) at Integrations 1 and 2.

Physiological data including heart rate variability (HRV), actigraphy, and sleep staging will be collected using a wearable device throughout the study.

#### Qualitative

Qualitative data will be collected in the form of semi-structured interviews at Prep 1, Integrations 1 and 2, Final Follow Up, and the 6-month Remote follow up (see **Table 4**). The aims of these interviews are to assess how they lived experience of fibromyalgia change after psychedelic-assisted therapy, as well as its potential therapeutic mechanisms. Patient reports often capture subtle yet powerful changes in personal narratives before and after psychedelic therapy that quantitative data are not able to record.

### Analysis strategy

Our two primary, mechanistic hypotheses relate to EEG and MRI:

H1 (EEG): We hypothesise an increase in signal complexity (LZc) at peak drug effects under psilocybin.
H2 (fMRI): we hypothesise a decrease in brain network modularity after PAT.

Secondary outcomes include:

Reduced alpha power under psilocybin.
A relationship between acute increases in LZc and post-PAT changes in psychological flexibility, measured by the BEAQ.
Changes in mass univariate functional connectivity after PAT.
Changes in PFC-tract diffusivity after PAT.
Changes in EEG ERP related markers of functional plasticity after PAT.
Changes in pain symptomatology after PAT, measured by the BPI-IS.

All secondary outcomes will be exploratory (see **Table 5**). Sub-acute EEG analyses will follow previously outlined protocols (91–93). With an assumption of high co-linearity between core outcomes we would explore data reduction approaches (e.g., factor or principal component analyses or canonical correlation analysis) to investigate key contrasts. Two tailed tests will be performed if findings are not aligned with prior hypotheses. Due to prior hypotheses (above) on directionality, one tailed t-tests will be appropriate to perform for H1 & H2. Multiple comparisons corrections and Bayesian analyses will be performed where deemed appropriate. Please see **Table 5** for timepoints for each analysis.

## IMP management

A Schedule 1 licence for possession and storage of psilocybin has been obtained from the UK Home Office. Psilocybin supplied by Usona Insitute. Manufacture and encapsulation will be performed by Lonza Pharma and Biotech. Good Manufacturing Practise (GMP) will be maintained at all stages of manufacture. The IMP will be stored in a secure safe at Imperial College London, Hammersmith Campus.

## Data management

Data will be managed as per the Imperial College Data Management Standard Operating Procedures and a study- specific data management plan. All data collection and management softwares have been meet GDPR standards and have been approved by Imperial College London.

## Dissemination

The results of this study will be published in academic journals and presented in both the academic and public domain, including at scientific conferences and in the media in public engagement forums. Patient confidentiality will be maintained in all the above. All publications and presentations relating to the study will be overseen by the P.I and C.I. Authorship of parallel studies initiated outside of the Study Co-ordination Team will be according to the individuals involved in the project but must acknowledge the contribution of the Study Coordination Team.

## Ethics and trial registration

This study has received a favourable opinion from the London Central Research Ethics Committee and is sponsored by Imperial College London’s Research Governance and Integrity Team. All participants will provide their written informed consent to be screened and, if relevant, participate in this study. The Medicines and Healthcare products Regulatory Agency (MHRA) has confirmed its status as a non-clinical trial and waived the need for MHRA approval. The study has been reviewed and approved by the Health Research Authority (HRA). The study protocol has undergone external peer review and was co-developed with patient advisors. All staff have undergone Good Clinical Practice (GCP) training. The study has been adopted by the National Institute of Health Research (NIHR) Clinical Research Network (CRN) and has been registered on clinicaltrials.gov (NCT05548075). All study sessions will take place at the NIHR-funded Imperial College Research Facility (ICRF) and Imperial Clinical Imaging Facility (CIF).

## Conclusion

By publishing the study protocol, we aim to improve methodological transparency, rigour, and accountability and subsequently contribute towards more impactful outcomes. We also aim to highlight the importance of patient involvement in protocol design in generating relevant and equitable research. This study will investigate effect of psilocybin on the neural mechanisms in a fibromyalgia population. The results will provide the first EEG recordings of the acute psychedelic state in a clinical population. Further, they may inform the viability of psilocybin as a potential treatment option and help shape subsequent clinical trials.

## Data availability statement

The original contributions presented in the study are included in the article/supplementary material. Further inquiries can be directed to the corresponding author.

## Author contributions

RCH: Conceptualization, Methodology, Project administration, Supervision, Writing – review & editing, Funding acquisition. JC: Conceptualization, Methodology, Project administration. Investigation, Writing – review & editing. JB: Conceptualization, Methodology, Project administration, Investigation, Writing – original draft, Writing – review & editing. DJN: Conceptualization, Supervision, Project administration, Writing – review & editing. KA: Conceptualization, Project administration, Investigation, Writing – review & editing. DE: Project administration, Investigation, Writing – review & editing. KG: Investigation, Writing – review & editing. LM: Investigation, Writing – review & editing. TB: Investigation, Writing – review & editing.
